# Resting State Networks in the TgF344-AD Rat Model of Alzheimer’s Disease Are Altered From Early Stages

**DOI:** 10.3389/fnagi.2019.00213

**Published:** 2019-08-08

**Authors:** Raúl Tudela, Emma Muñoz-Moreno, Roser Sala-Llonch, Xavier López-Gil, Guadalupe Soria

**Affiliations:** ^1^Consorcio Centro de Investigación Biomédica en Red (CIBER) de Bioingeniería, Biomateriales y Nanomedicina (CIBER-BBN), Group of Biomedical Imaging, University of Barcelona, Barcelona, Spain; ^2^Experimental 7T MRI Unit, Institut d’Investigacions Biomèdiques August Pi I Sunyer (IDIBAPS), Barcelona, Spain; ^3^Department of Biomedicine, Faculty of Medicine, University of Barcelona, Barcelona, Spain

**Keywords:** Alzheimer’s disease, animal model, magnetic resonance imaging, resting state, connectivity, independent component analysis, transgenic, rats

## Abstract

A better and non-invasive characterization of the preclinical phases of Alzheimer’s disease (AD) is important to advance its diagnosis and obtain more effective benefits from potential treatments. The TgF344-AD rat model has been well characterized and shows molecular, behavioral and brain connectivity alterations that resemble the silent period of the pathology. Our aim was to longitudinally investigate functional brain connectivity in established resting-state networks (RSNs) obtained by independent component analysis (ICA) in a cohort of TgF344-AD and control rats every 3 months, from 5 to 18 months of age, to cover different stages of the disease. Before each acquisition, working memory performance was evaluated by the delayed non match-to-sample (DNMS) task. Differences in the temporal evolution were observed between groups in the amplitude and shape of the somatosensorial and sensorimotor networks but not in the whole default mode network (DMN). Subsequent high dimensional ICA analysis showed early alterations in the anterior DMN subnetwork activity of TgF344-AD rats compared to controls. Performance of DNMS task was positively correlated with somatosensorial network at 5 months of age in the wild-type (WT) animals but not in the Tg-F344 rats. At different time points, DMN showed negative correlation with cognitive performance in the control group while in the transgenic group the correlation was positive. In addition, behavioral differences observed at 5 months of age correlated with alterations in the posterior DMN subnetwork. We have demonstrated that functional connectivity using ICA represents a useful biomarker also in animal models of AD such as the TgF344AD rats, as it allows the identification of alterations associated with the progression of the disease, detecting differences in specific networks even at very early stages.

## Introduction

Alzheimer’s disease (AD) is a progressive age-related neurodegenerative disease, which has become the most common form of dementia in elderly populations. A key point to find effective treatments is the understanding of the disease evolution from the very early stages, before the accumulation of amyloid plaques, neurofibrillary tangles and neural loss that extensively damage the brain. Evidences of early brain changes associated to AD have been suggested decades prior to its clinical diagnosis (Sperling et al., 2011; Vos et al., 2013; Dubois et al., 2016) but the late appearance of AD symptoms hinders the study of the disease progression in human cohorts. In this line, the use of transgenic animal models of AD is of great utility, especially in order to tackle the early and silent phases of the disease and to study its longitudinal progression (Leon et al., 2010; Do Carmo et al., 2013; Sabbagh et al., 2013; Galeano et al., 2014). The TgF344-AD rats represent one of the most suitable and promising animal models for AD research, as they manifest in an age-dependent manner all the AD’s pathological hallmarks: cerebral amyloidosis, taupathy, oligomeric Aβ, gliosis, apoptotic loss of neurons, and behavioral impairment, which represent a complete repertoire of AD pathological features (Cohen et al., 2013).

In the last 2 years, a wide number of important studies have confirmed not only the initial phenotype of TgF344-AD rats but also have expanded the knowledge regarding the silent period of the pathology in these animals. As early as 6 months of age, when soluble Aβ, hyper-p-tau, and gliosis are increased, but prior to reported hippocampal-dependent behavioral deficits (Cohen et al., 2013), basal synaptic strength in medial perforant path dentate granule cell was weakened and long-term potentiation was pathologically altered (Smith and McMahon, 2018). At the same early stage, accumulation of hyperphosphorylated tau was found in the locus coeruleus prior to tau pathology in the medial entorhinal cortex or Hippocampus (HPC; Rorabaugh et al., 2017). Additionally, TgF344-AD rats showed reduced locus coeruleus fiber density in the dentate gyrus and norepinephrine levels in the HPC, with no frank noradrenergic cell body loss (Rorabaugh et al., 2017). In a different study, TgF344-AD rats of 9 months exhibited significant cerebrovascular dysfunction dependent on vessel amyloid load and impaired theta-gamma phase-amplitude coupling, indicating neuronal network dysfunction in the early stage of tau and Aβ pathologies (Joo et al., 2017).

Early stage behavioral markers have been also found in these animals. For instance, TgF344-AD rats exhibited increased anxiety-like behavior, without significant deficits in the spatial memory at 4–6 months, when cerebral plaque burden is minimal (Pentkowski et al., 2018). Moreover, depressive-like behavior occurred earlier than cognitive deficits in TgF344-AD rats, consistent with AD in many patients (Voorhees et al., 2018). Interestingly, a neuroprotective compound, P7C3, chronically administered from 6 months blocked the acquisition of the early depressive-like behavior, later cognitive impairment, and ultimately neuronal cell loss in the TgF344-AD rat without altering amyloid deposition or indicators of neuroinflammation at early stages, (Voorhees et al., 2018). Using the Morris water task, clear spatial navigation impairments at 10–11 months of age were identified in TgF344-AD, although by 7–8 months of age these rats already displayed a significant decrease in the directness of their swim trajectories (Berkowitz et al., 2018). Altogether, these data demonstrate a correspondence to navigation alterations observed in individuals with preclinical or prodromal AD (Pai and Jacobs, 2004; Guariglia and Nitrini, 2009), suggesting additional translational validity for the TgF344-AD model.

In addition, AD has been defined as a disconnection syndrome, as described by several studies focused on brain connectivity (Delbeuck et al., 2003, 2007; Yong et al., 2009; Filippi and Agosta, 2011). Accumulation of Aβ and hyperphosphorylated tau, major hallmarks of AD, has been associated with complex disturbances in synaptic and neuronal function leading to impairments of coordinated activity in the neuronal networks that support memory and cognition (Selkoe, 2002; Palop and Mucke, 2016). In this line, altered cortical and hippocampal excitability was found in TgF344-AD rats relative to wild-type (WT) controls, together with increased rate of high-voltage spindles and decreased auditory sensory processing (Stoiljkovic et al., 2019). A recent report using scalp electroencephalography suggested altered phase synchronization between medial prefrontal cortex (mPFC) and HPC in AD patients (Hata et al., 2016). Interestingly, (Bazzigaluppi et al., 2018) reported reduced mPFC-HPC coherence in TgF344-AD rats suggesting GABAergic neuronal network dysfunctions at 9 months of age, that is, before the onset of significant cognitive symptomatology. Even before, at 6 months, minor functional deficits could be detected by functional magnetic resonance imaging (MRI) in the absence of microstructural changes including impaired connectivity with the HPC, cingulate and sensory regions (Anckaerts et al., 2019). Our group has also demonstrated important MRI based structural connectivity alterations in TgF344-AD rats at 5 months of age (Muñoz-Moreno et al., 2018). At a global level, structural networks showed lower integration and segregation in transgenic than in control rats, pointing to a different pattern of anatomical connections in subjects developing AD. Decreased functional network properties were observed among others in amygdala, VTA and insular cortex, regions related to reward, memory, and sensory performance, known to be altered in patients with AD or mild cognitive impairment (MCI; Muñoz-Moreno et al., 2018). This previous work was based in the analysis of connectivity between brain regions defined according to an atlas (Schwarz et al., 2006; Valdés-Hernández et al., 2011). Our objective in the present study was to longitudinally study functional brain connectivity in established resting-state networks (RSNs) obtained by independent component analysis (ICA). Opposite to the previously mentioned approach, ICA does not require a previous brain parcellation and allows the characterization of RSNs spatial location and magnitude of the functional activity (Nickerson et al., 2017), becoming one of the most popular techniques for the analysis of resting state functional magnetic resonance imaging (rs-fMRI). Indeed, it has been widely applied to the analysis of RSNs in prodromal or clinical AD phases, showing alterations mainly in the default mode, sensorimotor, salience and limbic networks (Greicius et al., 2004; Sorg et al., 2007; Agosta et al., 2012; Binnewijzend et al., 2012; Brier et al., 2012; Damoiseaux et al., 2012; Rami et al., 2012; Dennis and Thompson, 2014) and revealing the potential of this technique as an AD biomarker (Badhwar et al., 2017). Consistent with these findings, we hypothesized that RSNs in the TgF344AD rats would show time-dependent alterations revealing neuronal dysfunction in networks not explored to the moment in this promising animal model of AD, namely: default mode network (DMN), sensorimotor and somatosensory networks. In addition, we hypothesized that RSNs properties would have a relation with the cognitive outcome performed by these animals in the delayed non-matched to sample task.

## Materials and Methods

### Subjects

Experiments were longitudinally performed with two groups of male rats followed from 5 to 18 months of age: one group of TgF344-AD rats (Cohen et al., 2013) and another group of WT littermates (see Table 1). Both groups of rats were housed under controlled temperature (21 ± 10°C) and humidity (55 ± 10%), with a 12-h light/12-h dark cycle (light between 8:00 AM and 8:00 PM). Food and water were available *ad libitum* during all experiments, except the periods of behavioral training and testing, when they received only the 75% of their usual food intake. Animal work was performed according to local legislation (Decree 214/1997 of July 30th by the “Departament d’Agricultura, Ramaderia i Pesca de la Generalitat de Catalunya”) under the approval of the Ethics Committee (CEEA) at the University of Barcelona, and in compliance with European legislation.

**Table 1:** Group size and age (mean ± SD) of each experimental group at the five acquisition time points.

Group	Wild type			TgF344-AD		
Time	Group size	Age (days)	Age (months)	Group size	Age (days)	Age (months)
t1	6	158 ± 10	5.3 ± 0.3	8	189 ± 29	6.3 ± 1
t2	10	242 ± 10	8.1 ± 0.3	9	256 ± 8	8.5 ± 0.3
t3	9	339 ± 4	11.3 ± 0.1	9	338 ± 3	11.3 ± 0.1
t4	9	443 ± 8	14.8 ± 0.3	9	448 ± 6	14.9 ± 0.2
t5	9	534 ± 9	17.8 ± 0.3	6	543 ± 14	18.1 ± 0.5

### Cognitive Function Evaluation

The working memory performance was evaluated by means of the delayed non-matching-to-sample (DNMS) task, following a procedure previously explained in detail (Muñoz-Moreno et al., 2018). Briefly, animals underwent an habituation phase followed by six training phases before the DNMS task began. After the training period, the DNMS task was carried out before the MRI acquisitions every 3 months. All the stages took place in operant chambers (Med Associates, Fairfax, VT, USA) with a pellet dispenser and three retractile levers, two of them in the same side where the feeder is (namely right and left levers) and the other in the opposite side (center lever).

Once an animal achieved the acquisition of training phase criteria, the first DNMS task began. It required the animal to press the retractable lever presented on a random basis on the left or right (sample response) to initiate the trial. This began a delay phase of random duration between 1 s and 30 s. After the delay the animals had to press the center lever located on the opposite wall. Animals then returned to where both the left and right levers were extended (match/non-match phase). The correct response required a press on the opposite lever pressed during the previous sample phase (constituting the non-match response), which was followed by delivery of a food pellet into the hopper. An incorrect response (pressing the same lever as the one pressed in the sample phase) produced a 5 s time-out in which the overhead lights were turned off and no food pellet was delivered. Trials were separated by 10 s. Each session finished after 90 min or when 90 trials were completed.

At the first time point 15 DNMS test sessions (3 weeks, five sessions per week) were performed to evaluate and consolidate the task learned during the training phase. At each of the following four time points 10 DNMS test sessions (2 weeks) were performed before each MRI acquisition. The total number of trials and the ratio of correct responses were recorded in each session to assess the cognitive skills.

### Magnetic Resonance Imaging

MRI experiments were conducted at five time points (see Table 1) for both groups of rats on a 7 Tesla BioSpec 70/30 horizontal animal scanner (Bruker BioSpin, Ettlingen, Germany), equipped with an actively shielded gradients system (400 mT/m, inner diameter of 12 cm). The receiver coil was a 4-channel phased-array surface coil for the rat brain. Animals were placed in supine position in a Plexiglas holder with a nose cone for administering anesthesic gases (1.5% isofluorane in a mixture of 30% O_2_ and 70% CO_2_) and were fixed using a tooth bar, ear bars and adhesive tape. Then, the rat received a 0.5 ml bolus of medetomidine (0.05 mg/kg; s.c.) and a catheter was subcutaneously implanted in the back of the rat for continuous perfusion of medetomidine during the experiment. Isofluorane was gradually decreased until 0% and 15 min after the bolus the perfusion of medetomidine (0.05 mg/kg; s.c) started at a rate of 1 ml/h.

Localizer scans were used to ensure accurate positioning of the head in the magnetic isocenter. For anatomical reference T2-weighted images were acquired with a RARE (rapid acquisition with relaxation enhancement) sequence, with effective echo time TE = 35.3 ms, TR = 6,000 ms and RARE factor 8. Matrix size was 256 × 256 with an in-plane voxel size of 0.1172 × 0.1172 mm^2^, 40 slices, slice thickness 0.8 mm, resulting in a field of view (FOV) of 30 × 30 × 32 mm^3^.

Resting state functional MRI was acquired using a single-shot gradient echo planar imaging (EPI) T2* sequence. Six-hundred volumes of 64 × 64 × 34 voxels with a size of 0.4 × 0.4 × 0.6 mm^3^/voxel (FOV of 25.6 × 25.6 × 20.4 mm^3^) were acquired with TR = 2,000 ms and TE = 10.75 ms.

### Resting State fMRI Data Processing

Resting-state preprocessing included slice timing, motion correction by spatial realignment using SPM8^1^ and correction of EPI distortion by elastic registration to the T2-weighted volume using ANTs (Avants et al., 2008). As explained in Muñoz-Moreno et al. (2018), a brain mask obtained from a rat brain atlas was registered from T2 to the preprocessed mean resting-state volume to skull-strip the subject volume. After applying the brain mask to all the volumes, they were registered to the atlas template using elastic registration.

The five first volumes of each registered resting state acquisition were discarded to ensure the magnetization reached the steady state. Spatial noise was reduced in all volumes by using FSL SUSAN filter (Smith and Brady, 1997) with FWHM = 1.2 mm followed by a temporal detrending, and regression of motion parameters using Nilearn (Abraham et al., 2014). Finally, we applied *z*-score normalization and a band-pass filtering between 0.01 and 0.1 Hz with a Hamming window filter implemented in NiTime^2^. The processed acquisitions of the whole cohort were used to obtain the brain network components with the multisession temporal ICA concatenated approach implemented in FSL’s MELODIC (Beckmann and Smith, 2004, 2005; Beckmann et al., 2005). The analysis was set to extract 30 independent components from the rs-fMRI data (ICA_30_). Based on their relevance to functional alterations related to aging and AD (Agosta et al., 2010, 2012; Stephen et al., 2010; Binnewijzend et al., 2012; Brier et al., 2012; Damoiseaux, 2017), the selected components were the somatosensorial, the sensorimotor and the DMNs. The anatomical structures comprised in these networks were selected by visual inspection of their spatial maps based on previous studies (Henckens et al., 2015; Sierakowiak et al., 2015; Bajic et al., 2016; Hsu et al., 2016).

### Analysis of the Somatosensorial, Sensorimotor and Default Mode Networks

Dual regression was performed to find the subject-specific time-series and individual spatial maps for the selected IC’s (Nickerson et al., 2017). For each RSN at each subject and each time point, we computed its amplitude and shape. Amplitude was defined as the standard deviation of the time-series obtained from the first stage of the dual regression. The component shape for each subject was computed as the mean of the *z*-values within the thresholded (*z* > 2.3) spatial map obtained from the second stage of the dual regression, where the differences in the spatial pattern, known to be related to the spatially distributed nature of correlations, can be observed (Nickerson et al., 2017).

Age and group effects in the temporal evolution of shape and amplitude were evaluated by means of linear mixed effect (LME) models (Oberg and Mahoney, 2007). LME models include parameters common to the entire sample (fixed effects as age or group) and subject specific parameters (random effects as the deviation from the population). Two LME models were considered to regress amplitude and shape respectively as a function of the age, group and group-age interaction as independent variables:

$$y_{s}=\beta_{0}+\beta_{1}\cdot \text{group+}\beta_{2}\cdot \text{age}+\beta_{3}\cdot \text{group}\cdot \text{age}+\beta_{4,\text{s}}+\xi \;s=1,\ldots ,N$$

where *y*_s_ is the amplitude or the shape of the subject s at a determined age. *β*_0_ is the global intercept, *β*_1_ is the fixed parameter for the group effect, *β*_2_ is the parameter for the age effect, and *β*_3_ the parameter for the influence of the interaction between age and group. *β*_4,s_ is the subject specific correction and ξ the regression error. Group and age effects and the interaction between them were considered significant when *p* < 0.05. In a second step, when a significant interaction appeared, control and transgenic groups were modeled separately, considering the age effect as independent variable.

Besides the LME analysis, the differences in amplitude and shape between groups at each time point were statistically evaluated using Kruskal–Wallis tests. We accounted for multiple comparison correction using the Benjamini–Hochberg false discovery rate (FDR), considering a statistical tendency if corrected *p* < 0.1, and significant difference if corrected *p* < 0.05.

To account for connectivity between networks, Pearson correlation between the time-series of pairs of networks were calculated for each subject and time point and the differences were statistically evaluated as it was done with the amplitude and shape. A similar LME model was also used to fit the correlation between networks as a function of group, age and their interaction.

Finally, to study relationship with cognition, the Spearman correlation coefficient was computed between the cognitive results and the amplitude and shape indices of the selected RSNs for the two groups and the five time points. Correlation was considered significant if *p* < 0.05.

### Subnetworks of the Default Mode Network

To identify patterns of subnetworks comprised within the DMN we ran a second ICA with 150 components (ICA_150_). A spatio-temporal criterion that included the temporal correlation of IC time-series and the spatial overlap between the IC maps was applied to identify which components of the ICA_150_ belonged to the DMN. Components from the ICA_150_ with high temporal correlation (*r* > 0.4) and high spatial overlap (more than 250 voxels in common) with the DMN were identified as subparts of this network. Amplitude and shape of the subnetworks were computed, and the connectivity within the DMN was evaluated with the pair-wise correlations between the timeseries of the subnetworks. The relationship with cognition was also evaluated with the DMN subnetworks using the Spearman correlation as mentioned above.

## Results

### Resting State Functional Networks

**Figure 1:**
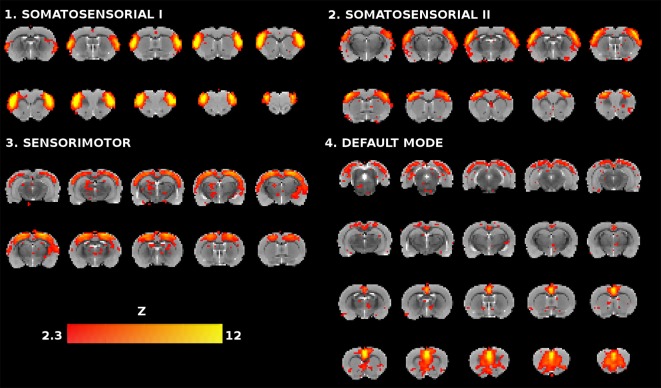
Coronal slices of the rat brain for the four selected independent component analysis (ICA) components (*Z* > 2.3). Each component is represented with 10 slices except for the default mode network (DMN), which has 20 slices, as it is a more extensive network and their slices serve as anatomical reference for the relative location of the other three components.

Based on the anatomical and functional relevance to AD, aging and the cognitive task, the analysis was focused on somatosensory, sensorimotor and DMN networks. From ICA_30_ four components were identified representing these functional networks (Figure 1). The somatosensorial network was represented by two IC’s, so called somatosensorial I and II. The spatial map of somatosensorial I network included primary somatosensory areas of the cortex related to upper limbs, jaw and oral surface of the limbs, while the anatomical distribution of somatosensorial II network covered primary barrel field cortex and secondary somatosensory areas. Motor cortex I and II were partially represented at the frontal part of these ICs’ (Moore et al., 1999; Menzel and Barth, 2005; Henckens et al., 2015; Ebbesen et al., 2017).

The third component was the sensorimotor mainly covering motor regions with a dorsoposterior preference, but with some overlapping in sensory cortical areas (Henckens et al., 2015; Sierakowiak et al., 2015). Finally, the DMN is characterized in rats by the activation of: (1) the medial prefrontal cortex, the most important association cortical area in the rat, showing connections with a great number of cortical and subcortical structures, responsible for decision making, planning of the actions and working memory functions; (2) the cingulate cortex, playing a critical role in stimulus-reinforcement learning and reward-guided selection of actions; and (3) the retrosplenial cortex involved in a variety of cognitive tasks including memory, navigation, and prospective thinking (Hamani et al., 2014; Sierakowiak et al., 2015; Bajic et al., 2016; Hsu et al., 2016).

### Amplitude and Shape of the Functional Networks

LME models were fitted to evaluate the longitudinal effects of age, group or their interaction in the RSN amplitude and shape. Table 2 shows the significant *p*-values of the LME coefficients for the model fitted to all the subjects and the models for control or TgF344-AD groups separately. Significant effect of age and group interaction was observed in the two somatosensorial networks. When each group was evaluated independently, a significant age effect in the somatosensorial I network for the control group and in the somatosensorial II network for the TgF344-AD group was found. This revealed the different temporal evolution observed in the amplitude and shape of the somatosensorial I and II networks between groups: while in controls amplitude and shape increased with age, the opposite effect was observed in the transgenic animals (Supplementary Figure S1).

**Table 2:** *p*-values of the linear mixed effect model coefficients; significance of group, age and its interaction in the model fitted to all subjects; significance of age effect in the group models.

	Network	All subjects			Control Age	TgF344-AD Age
		Group	Age	Group × Age		
Amplitude	Somatosensorial I	0.043	0.003	0.002	0.005	n.s.
	Somatosensorial II	0.038	n.s.	0.001	0.047	0.013
	Sensorimotor	n.s.	n.s.	n.s.	-	-
	Default Mode	n.s.	n.s.	n.s.	-	-
Shape	Somatosensorial I	n.s.	0.004	0.004	0.004	n.s.
	Somatosensorial II	n.s.	n.s.	0.021	n.s.	0.013
	Sensorimotor	n.s.	n.s.	n.s.	-	-
	Default Mode	n.s.	n.s.	n.s.	-	-

*Significance if p < 0.05*.

**Figure 2:**
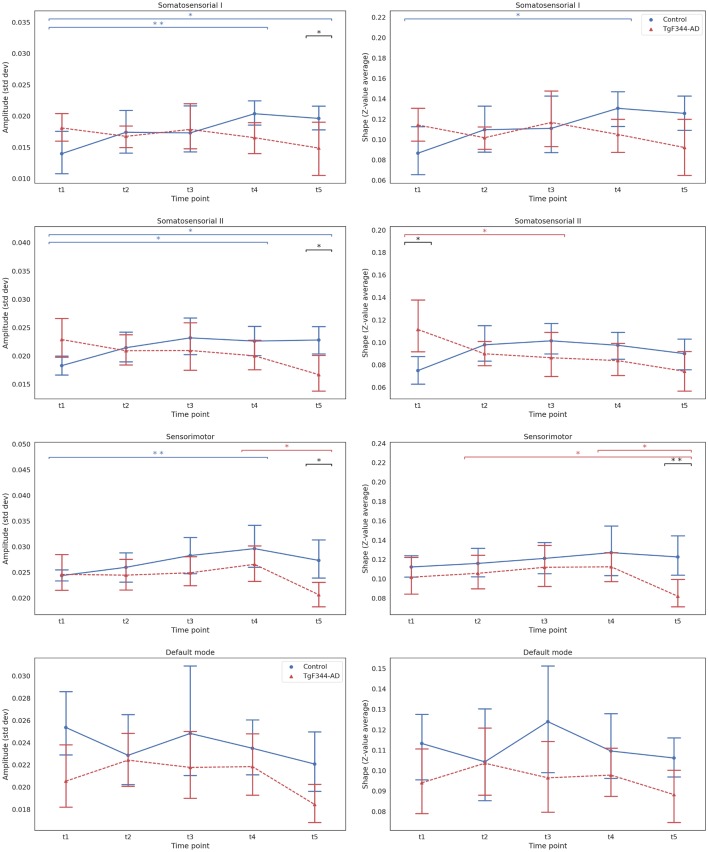
Mean and 95% confidence interval showing the evolution with time of the amplitude (left column) and the shape (right column) for each group and time point for the four selected independent components. **Indicates significant difference (*p*_FDR_ < 0.05), *a tendency to differences (*p*_FDR_ < 0.1) between groups or time points connected by the line under it. Blue for the wild type (WT) group and red for the TgF344-Alzheimer’s disease (AD) group, black lines for the differences between groups.

Subsequent analyses to compare the effect of genotype in shape and amplitude at each timepoint were performed by Kruskal–Wallis tests with FDR correction. Figure 2 shows the mean and the 95% confidence interval of the amplitude and shape of each network at each time point. For each network, amplitude and shape showed similar trends. The main differences between groups were observed at t5 in the sensorimotor and somatosensorial I and II networks. At this time point, TgF344-AD animals showed a higher fall in amplitude and shape values compared to the controls. Significant difference (*p* < 0.05) was only found in the shape of the sensorimotor network, although a tendency to significance (*p* < 0.1) was observed in the amplitude of somatosensory I and II and sensorimotor networks. Interestingly, there was a tendency for higher shape values in the transgenic group compared to controls in the somatosensorial II network at 5 months (t1).

Regarding longitudinal evolution, we confirmed the results obtained with the LME models: an overall increase of amplitude and shape measurements over time in the control group, while these measurements decreased in the TgF344-AD group. This behavior was clearly observed in the somatosensorial I network, where the amplitude values significantly increased between t1 and t4 in the control group (*p* < 0.05). The same tendency was observed in the somatosensorial II network, with increased amplitude at t4 and t5 compared with t1 (*p* < 0.1). On the other hand, the values of this network for the transgenic group descended, with a clear tendency in the shape between t1 and t3 (*p* < 0.1). In the sensorimotor network there was a significant increase in the amplitude in the control group between t1 and t4 (*p* < 0.05), while the values of amplitude and shape for the transgenic group remained more stable until the last point, where there was a tendency to fall between t4 and t5 (*p* < 0.1), breaking the linearity of the longitudinal trajectory. No clear tendency was observed in the DMN evolution, which seemed to have a different longitudinal behavior than the other RSNs studied.

The between-network connectivity of the four RSNs was calculated and analyzed by LME models. Although there were not significant differences between groups for any specific time point, the variation of the correlation with age between networks was higher in the control group. This information can be found in the Supplementary Figure S2.

### DMN Subnetworks

**Figure 3:**
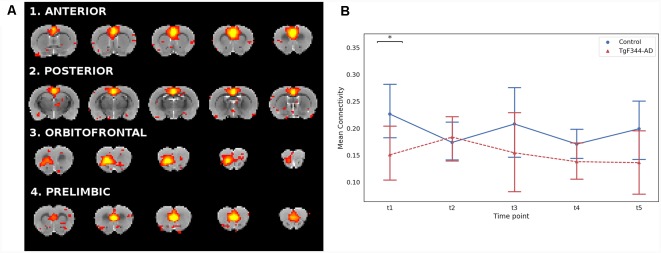
**(A)** Coronal slices of the rat brain for the four DMN subnetworks (*Z* > 2.3). Each component is represented with its most representative five slices. The color scale and the anatomical reference are the same as in Figure 1. **(B)** Mean connectivity and 95% confidence interval within the DMN for each group and each time point. *Indicates significant difference (*p* < 0.05) between groups . Blue lines for the WT group and red for the TgF344-AD group.

Given the well-established relationship between DMN connectivity and AD (Greicius et al., 2004; Sorg et al., 2009; Sperling et al., 2010), we analyzed the DMN subnetworks obtained from a high dimensional ICA. By using the spatio-temporal correspondence, 4 DMN subnetworks were identified within the ICA_150_ set: anterior, posterior, orbitofrontal and prelimbic (Figure 3A). The DMN overall mean connectivity, defined as the average correlation within all the DMN subnetworks, was significantly lower in the TgF344-AD rats compared to controls at the first time point (Figure 3B). In addition, we evaluated connectivity between pairs of subnetworks (Supplementary Figure S3) and we found a significant decrease of connectivity between the anterior and posterior DMN subnetworks at t4 in the transgenic animals compared to controls.

**Figure 4:**
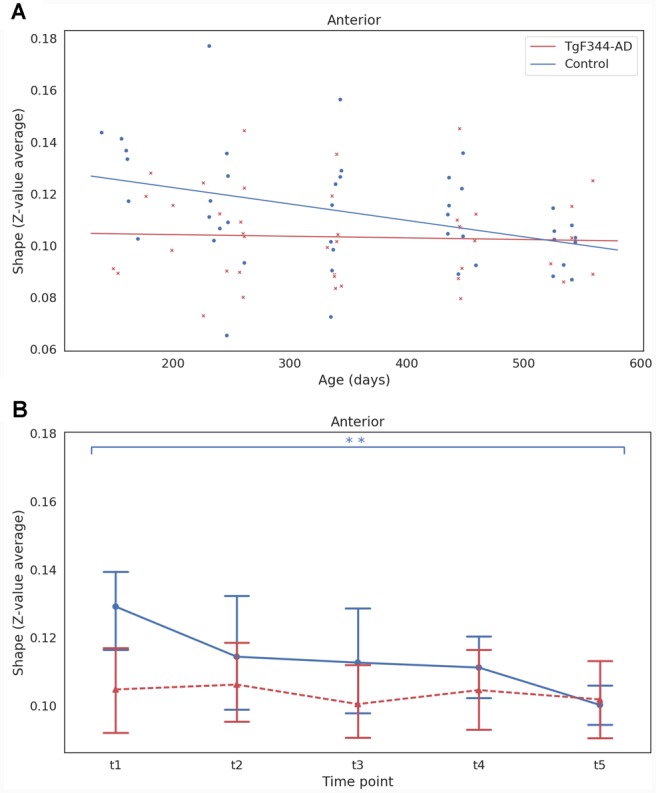
**(A)** Linear mixed effect (LME) model fit of the shape as function of age and group for the anterior DMN subnetwork. Each dot represents the shape of one animal at one time point. **(B)** Mean and 95% confidence interval showing the evolution with time of the shape for each group and time point for the anterior DMN subnetwork. **Indicates significant difference (*p*_FDR_ < 0.05) between groups or time points connected by the line under it. Blue for the WT group and red for the TgF344-AD group.

Amplitude and shape were also computed for each subject and time point for the DMN subnetworks. An LME model was adjusted for each subnetwork following the procedure used with the ICA_30_ networks. Significant linear effects of group (*p* = 0.015) and age (*p* = 0.003) were found in the shape of the anterior DMN subnetwork (Figure 4A). A significant decrease with age (*p* = 0.023) was observed in the amplitude of the same subnetwork. Figure 4B shows the mean and the 95% confidence interval of the shape of anterior DMN subnetwork. There was a clear decrease of shape in the control group, with a significant difference found between 5 and 18 months, while shape values of TgF344-AD animals were lower and maintained over time, suggesting dissimilarities in the anterior DMN subnetwork at early ages between both groups.

### Cognitive Function and Functional Networks

**Figure 5:**
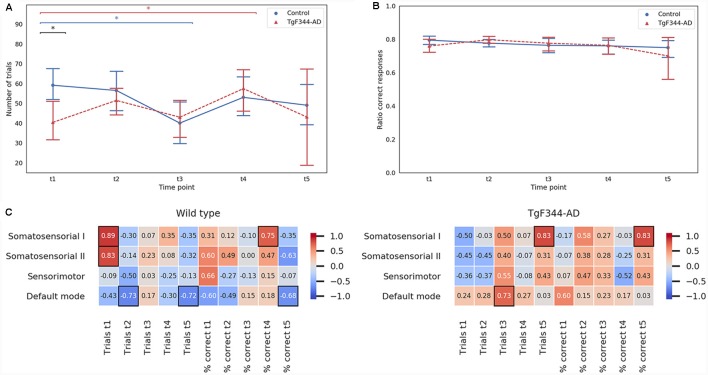
Mean and 95% confidence interval for **(A)** the number of trials and **(B)** the ratio of correct responses in the delayed non match-to-sample (DNMS) task performed by both control and TgF344-AD groups at the five time-points. *Indicates significant difference (*p* < 0.05) between groups connected by the line under it. Blue lines for the WT group and red for the TgF344-AD group. **(C)** Spearman correlation coefficients between each network amplitude and the number of trials and ratio of correct responses of the DNMS test at the five time-points for the control (right column) and the TgF344-AD (left column) groups. Black boxes indicate significant Spearman correlation (*p* < 0.05).

Cognitive function was evaluated by DNMS task, a working memory test. The number of trials and the ratio of correct responses for each time point are plotted in Figures 5A,B, respectively. At 5 months (t1), the transgenic group performed significantly less trials than the control group. However, this difference was not maintained over time. Subtle temporal differences were observed between genotypes. For instance, controls showed a significant decrease in the number of trials between 5 and 11 months while the transgenic group showed a significant increase between 5 and 15 months. Indeed, a clear increase of variability was observed at the last time point, 18 months, especially in the transgenic group, which could also be observed in the ratio of correct responses (Figure 5B).

In order to study if the RSN features had an impact on cognitive outcome, we calculated the Spearman correlation coefficients between RSN measures and the results of the cognitive test. Similar results were obtained with both amplitude and shape. For the sake of simplicity, in Figure 5C only the amplitude results are shown (for shape correlations see Supplementary Figure S4). For the WT group, the correlation between the number of trials performed in the DNMS and the amplitude showed significant positive correlations (Spearman’s *p* < 0.05) in the somatosensorial I and II networks at 5 months, and negative correlations (Spearman’s *p* < 0.05) were found at 8 and 18 months in the DMN. For the TgF344-AD group, the amplitude showed significant correlations (Spearman’s *p* < 0.05) in the DMN and the somatosensorial I at 11 and 18 months respectively. The percentage of correct responses showed, for the control group, a significant positive correlation with the somatosensorial I network at 13 months and a significant negative correlation with the DMN at 18 months. Meanwhile, in the TgF344-AD group a high correlation was observed between the percentage of correct responses and the somatosensorial I network at 18 months (Spearman’s *p* < 0.05). In general, the values of correlation between the amplitude and the cognitive results were lower for the control group than for the TgF344-AD group in the DMN.

Correlations of cognitive performance and DMN subnetwork measures are shown in Supplementary Figure S5. Very briefly, WT animals showed only significant negative correlations (orbitofrontal and posterior at 5 and 8 months, respectively, and anterior and prelimbic at 18 months). On the other hand, the TgF344-AD group showed positive significant correlations (posterior at 5 months, prelimbic at 8 months and anterior at 11 months), while only a significant negative correlation was observed at 15 months in the orbitofrontal subnetwork.

## Discussion

The study of RSN obtained with ICA has been suggested as a powerful AD biomarker (Badhwar et al., 2017). In order to provide relevant and highly translational tools in the preclinical AD field, our aim was to longitudinally investigate RSN functional connectivity in the novel and very promising animal model of AD, the TgF344-AD rat (Cohen et al., 2013). The study was performed by acquiring rs-fMRI in transgenic and control groups of rats at five time points from 5 to 18 months of age, to cover different stages of the disease. Before each acquisition working memory, performance was evaluated with DNMS task. In this study, we have shown different functional connectivity over time in somatosensorial, sensorimotor and the anterior DMNs in TgF344-AD rats compared to WT animals. Some of these network features, shape and amplitude, were associated to cognitive outcome as assessed by DNMS task.

Differences between groups in the temporal evolution were observed in the amplitude and shape of the somatosensorial I and II networks. While in control animals amplitude and shape increased with age, an opposite behavior was observed in the transgenic animals. This was confirmed by the significant increase of amplitude observed at 15 and 18 months vs. 5 months in WT animals. On the other hand, transgenic animals showed a decline of ICA metrics, significant between 15 and 18 months of age, especially in the sensorimotor network, but also in the somatosensorial II between 5 and 12 months. At the last time point, significant differences between groups were observed in these 3 networks, suggesting an additive effect of pathology and aging with more severe consequences for the TgF344-AD rats. Of particular interest are the significant higher shape values of transgenic animals compared to WT found in the somatosensorial II at 5 months, when no overt signs of Aβ deposition, neurofibrillary tangles, neuronal loss or memory impairment have been described yet in these animals (Cohen et al., 2013). In this line, a transitory increase of functional connectivity in sensorimotor areas has been observed in the course of prodromal AD in humans (Agosta et al., 2010) and also in the McGill-R-Thy1-APP transgenic rat (Parent et al., 2017). Thus, our results obtained at 5 months might be related to the hypothesized compensatory process in the setting of early pathology state induced by the initial associated biochemical alterations, or to the direct result of the early pathophysiological process of AD (Agosta et al., 2010). Indeed, (Cohen et al., 2013) and other preclinical studies have reported early signs of pathology around 6 months of age in the TgF344-AD rats. Hyperphosphorylated tau was detected in the locus coeruleus prior to accumulation in the medial entorhinal cortex or HPC (Rorabaugh et al., 2017). By using electrophysiological techniques, age-dependent disruption of elicited hippocampal oscillations in anesthetized TgF344-AD rats has been shown starting at 6 months of age (Stoiljkovic et al., 2019). Moreover, at the same age a decrease in basal synaptic strength has been observed at medial perforant path-dentate granule cell-synapses and long-term potentiation is pathologically altered, inducing early glutamate receptors dysfunction (Smith and McMahon, 2018). Thus, our early differences found in the somatosensorial II network might be a reflect of this initial pathophysiological process.

Alterations in the DMN have been classically linked not only to AD progression (Greicius et al., 2004; Buckner et al., 2005; Damoiseaux et al., 2012; Hohenfeld et al., 2018) but also to its initial phases (Binnewijzend et al., 2012). Indeed, the major accumulation of beta-amyloid plaques overlaps with the topography of DMN, suggesting the possibility that activity within the network may facilitate disease processes (Buckner et al., 2005) and reinforcing the importance of studying this network also in animal models of AD. Thus, given the well-known role of DMN in AD, one of our objectives was to longitudinally characterize its functional connectivity in the TgF344-AD rat. Unexpectedly, ICA metrics, amplitude and shape, did not show significant differences between WT and transgenic animals at any of the studied time points and no clear tendency was neither observed over time in any of the genotypes. However, as in humans (Buckner et al., 2008; Andrews-Hanna et al., 2010; Whitfield-Gabrieli et al., 2011; Veldsman et al., 2017), the rat DMN is composed of highly connected anatomical and functional subnetworks, which show different modulation in association with age-related cognitive dysfunction (Hsu et al., 2016). DMN decomposition in subnetworks, for example using high-dimensional ICA, is very promising to better localize functional connectivity alterations in AD (Dipasquale et al., 2015). Sparse literature is available regarding DMN subnetworks in the rat. Lu et al. (2012) demonstrated the rat DMN and the subsequent network modularity analysis identified two distinct subsystems: a temporal-prefrontal subsystem centered on the prefrontal cortex including the cingulate, orbitofrontal, and prelimbic cortex, with strong functional correlation with the temporal cluster of primary sensory cortices, and another subsystem centered in the retrosplenial cortex showing strong functional correlation with HPC, posterior parietal cortex, and the secondary visual cortex. Accordingly, a recent study fractionated the DMN into an anterior and a posterior subsystem, further segregated into five modules (Hsu et al., 2016). After the high dimensional ICA_150_, four subcomponents were identified within the DMN that partially agree with those described in the previous literature. For instance, the anterior subnetwork, including the cingulate cortex, and the posterior subnetwork, including the retrosplenial cortex, overlap with the frontal and the retrosplenial modules from Hsu et al. On the other hand, the orbitofrontal and prelimbic subcomponents of DMN we describe in Figure 3 would be included in the frontal module of the same study. The main difference comparing our DMN subcomponents with those previously described is the lack of hippocampal representation, probably due to different likelihood parameters used in this work compared to the aforementioned (Lu et al., 2012; Hsu et al., 2016). Nonetheless, the overall mean connectivity within and between the 4 subnetworks was significantly lower in TgF344-AD rats compared to control animals at 5 months revealing decreased cohesion in the transgenic group. Moreover, there was a different temporal evolution of shape in the anterior subnetwork which significantly decreased over time in the WT animals while it remained low in transgenic animals. This result suggests early alterations in the anterior DMN activity of TgF344-AD rats and is in agreement with clinical data in patients, showing connectivity reductions in the posterior-DMN (Koch et al., 2015), with altered anterior-posterior DMN connectivity (Jones et al., 2012; Song et al., 2013). Moreover, earlier in the disease, regions of the posterior-DMN start to disengage whereas regions within the anterior and ventral networks enhance their connectivity. However, as the disease progresses, connectivity within all systems eventually deteriorates (Damoiseaux et al., 2012). Regarding other animal models of AD, increased prefrontal-hippocampal network synchronicity in 3-month-old APPNL−G−F mice has been shown before prominent amyloid plaque deposition (Latif-Hernandez et al., 2019). As previously postulated, early functional deficits preceding the onset of fibrillary Aβ are possibly related to synaptic vulnerabilities, which supports the concept that localized Aβ deposition may be dependent on the default patterns of activity preceding disease onset (Buckner et al., 2005; Parent et al., 2017). In addition, neurovascular dysfunction in prefrontal areas of the cortex (Joo et al., 2017; Bazzigaluppi et al., 2018) and reduced functional connectivity throughout the brain of TgF344-AD rats have been described at the age of 10 months (Anckaerts et al., 2019). In this line, our study represents the first evidence of early DMN alterations in the TgF344-AD rat, giving to this animal model a remarkable characteristic as translational AD model, since DMN is considered the key RSN in AD progression (López-Sanz et al., 2017).

Coupled to longitudinal MRI scans, working memory of rats was evaluated by the DMNS test (Dunnett et al., 1988). This task, widely used in animals and also in humans, requires subjects to hold a visual stimulus “on line” over a delay interval before responding to a choice of stimuli, in our case the novel stimulus. DNMS is sensitive to subtle changes in hippocampal neurophysiological dynamics that may not be visible in behavior until later in life (Callaghan et al., 2012; Hok et al., 2012). Indeed, DNMS a benchmark test of rule learning and recognition memory, is a complex cognitive task involving aspects of executive functions such as abstraction, cognitive flexibility, category shifting as well as conditional associative learning and working memory (Moore et al., 2012), which requires an appropriate functioning of the brain networks. In this line, we correlated the properties of RSN with two different behavioral outcomes: trials to criteria and percentage of correct responses. Somatosensorial networks including sensory areas of the cortex and anterolateral motor regions is required for responding to external stimuli playing a crucial role especially during the DNMS training period where rats have to associate visual cues with sensory reward responses. At 5 months of age, control animals showed high correlation between the number of trials to criteria to achieve the DNMS task and amplitude and shape of both somatosensorial networks. However, such correlation was not observed in transgenic animals, which performed fewer number of trials than the controls suggesting early alterations of this RSN in transgenic animals. In addition, amplitude of somatosensorial I network was strongly correlated with both behavioral outcomes at the latest time-point, 18 months. At this moment, the variability of DNMS performance increases considerably pointing again to its direct dependence on the proper functioning of the network.

Regarding DMN, we found a solid negative correlation with the cognitive task in WT animals, especially at 8 and 18 months. Not only this was undetected in the TgF344-AD rats but a strong positive correlation with the number of trials at 12 months (t3) was observed for this group. When we investigated the relation with the subcomponents of the DMN (Supplementary Figure S5) we found that in WTs the negative correlation at 5 months was linked to the orbitofrontal node while at 18 months it was to the anterior node. Interestingly, the behavioral differences observed at 5 months of age were correlated with alterations in the posterior subnetwork of the DMN in line with results from AD patients (Damoiseaux et al., 2012; Koch et al., 2015). All these findings support the hypothesis that in TgF344-AD rat model, as in other AD animal models and also in AD patients, there are alterations in somatosensory and DMN networks which have a direct impact in the cognitive and behavioral outcome and which appear before Aβ accumulation.

Although DMNS task gives a global measure of working memory and has been extensively validated in animal studies (Dunnett et al., 1988; Callaghan et al., 2012) it has some limitations. First, the acquisition phase of the DNMS task serves as a test of rule learning and transgenic animals took more time to finish the training period arriving later to the DNMS task (Muñoz-Moreno et al., 2018). Thus, we cannot rule out the supposition that the differences found in the RSN at 5 months may be related to their initial cognitive level. Another limitation of the study is the intensive and long testing periods during the whole longitudinal study. Namely, almost 2 months of training phase plus 10 days of DNMS testing every 3 months from 5 to 18 months. Since no important differences were found in the DMNS task until the increase of variability observed at 18 months in TgF344-Ad rats, we hypothesize that the repetition of DNMS task, during 10 days every 3 months may help to maintain the cognitive performance, at least until 15 months. This type of cognitive intervention might modify the evolution of functional networks and their connections in the transgenic rats as a compensatory mechanism at least during the intermediate stages. The fact that most significant differences between RSNs of transgenic and control animals were found in the earliest or the latest time points supports this hypothesis. Gender bias in neuroscience and preclinical studies is an issue to take into account (Beery and Zucker, 2011) and for this reason further experiments with additional experimental groups including males and females with and without DMNS testing are needed to fully demonstrate this idea.

## Conclusion

In summary, we have demonstrated that functional connectivity analysis using ICA represents a useful biomarker also in animal models of AD such as the TgF344AD rats, as it allows the identification of alterations associated with the progression of the disease, detecting differences in specific networks even at very early stages. The evolution of the functional networks in the TgF344-AD reproduced some of the disconnection syndrome aspects observed in humans (Agosta et al., 2010; Damoiseaux et al., 2012; Dennis and Thompson, 2014; Dipasquale et al., 2015), reinforcing its validity as an AD model. Moreover, for the first time to our knowledge, subnetworks of the DMN were demonstrated in a rodent model of AD revealing early decreased network cohesion, in agreement with previous studies in AD patients (Dipasquale et al., 2015); and finally, our work suggests that periodic DMNS testing might induce cognitive compensation in the TgF344-AD rats compared to controls in order to maintain the global brain function, especially during the middle stages of the disease.

## Data Availability

The datasets generated for this study are available on request to the corresponding author.

## Ethics Statement

### Animal Subjects

The animal study was reviewed and approved by Ethics Committee (CEEA) at the University of Barcelona.

## Author Contributions

All authors were involved in the conception and design of the project and manuscript writing and editing. Besides, GS was involved in data acquisition and data interpretation, XL-G was in charge of the animal caring and cognitive training and evaluation. RT, EM-M and RS-L were involved in the data processing and analysis and data interpretation. RT also performed the ICA analysis and cognitive correlation. RT, EM-M and GS were the major contributors in writing the manuscript. All authors read and approved the final manuscript.

## Conflict of Interest Statement

The authors declare that the research was conducted in the absence of any commercial or financial relationships that could be construed as a potential conflict of interest.

## Footnotes

^1^www.fil.ion.ucl.ac.uk/spm

^2^http://nipy.org/nitime/

## Supplementary Material

FIGURE S1 Linear mixed effect model fit of the amplitude (right column) and the shape (left column) as function of age and group. Blue for the control group, red for the TgF344-AD group. Each dot represents the amplitude or shape of one animal at one time point.

FIGURE S2 Correlation between the time series of each network. Each graph presents the average correlation between the four functional networks for each time point (t1–t5), in the lower triangle the results for the wild type group and in the upper triangle for the TgF344-AD group.

FIGURE S3 Mean connectivity between the four DMN subnetworks (anterior, posterior, orbitofrontal and prelimbic) at each time point (t1–t5). In the lower triangle the results for the wild type group and in the upper triangle for the TgF344-AD group. Black boxes indicate a difference (*p* < 0.05) between groups.

FIGURE S4 Spearman correlation coefficients between each network shape and the number of trials and ratio of correct responses of the DNMS test at the five time-points for the wild type (right column) and the TgF344-AD (left column) groups. Black boxes indicate significant Spearman correlation (*p* < 0.05).

FIGURE S5 Spearman correlation coefficients between DMN subnetworks amplitude (top row) or shape (low row) and the number of trials and ratio of correct responses of the DNMS test at the five time-points for the wild type (right column) and the TgF344-AD (left column) groups. Black boxes indicate significant Spearman correlation (*p* < 0.05).

## Abbreviations

AD: Alzheimer’s disease
DMN: Default mode network
DNMS: Delayed non-matching-to-sample
EPI: Echoplanar imaging
FOV: Field of view
FWHM: Full width half maximum
ICA: Independent component analysis
LME: Linear Mixed Effects
MCI: Mild Cognitive Impairment
MRI: Magnetic resonance imaging
RARE: Rapid acquisition with relaxation enhancement
rs-fMRI: Resting state functional magnetic resonance imaging
RSN: Resting state network
TE: Echo time
TR: Repetition time.
