# Supplementary figures and images for: Resting State Networks in the TgF344-AD Rat Model of Alzheimer’s Disease Are Altered From Early Stages
Source: Front Aging Neurosci. 2019 Aug 8;11:213. doi: 10.3389/fnagi.2019.00213 (PMC6694297; doi:10.3389/fnagi.2019.00213)

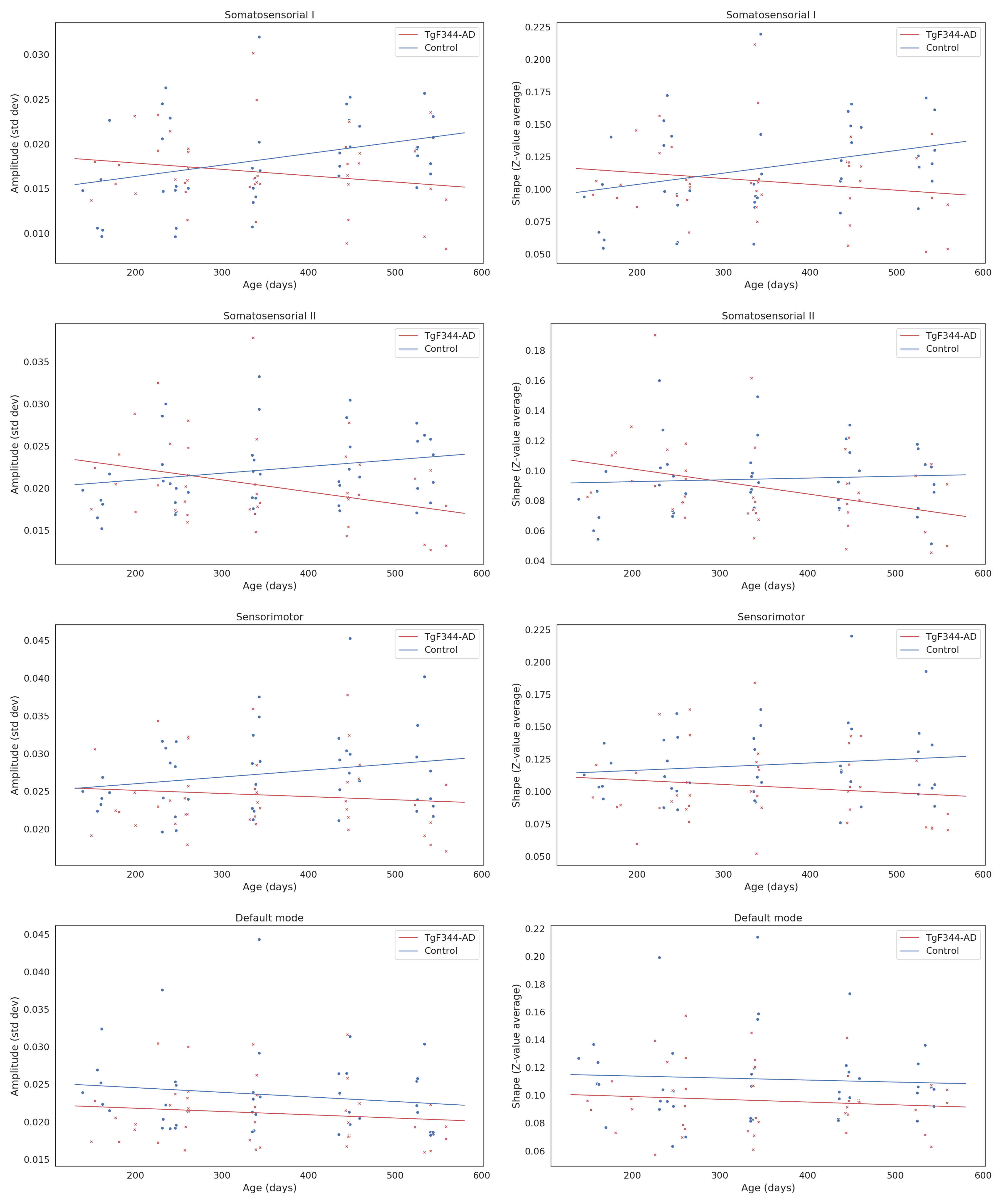

Supplement: FIGURE S1 — Linear mixed effect model fit of the amplitude (right column) and the shape (left column) as function of age and group. Blue for the control group, red for the TgF344-AD group. Each dot represents the amplitude or shape of one animal at one time point. [file Image_1.TIF]

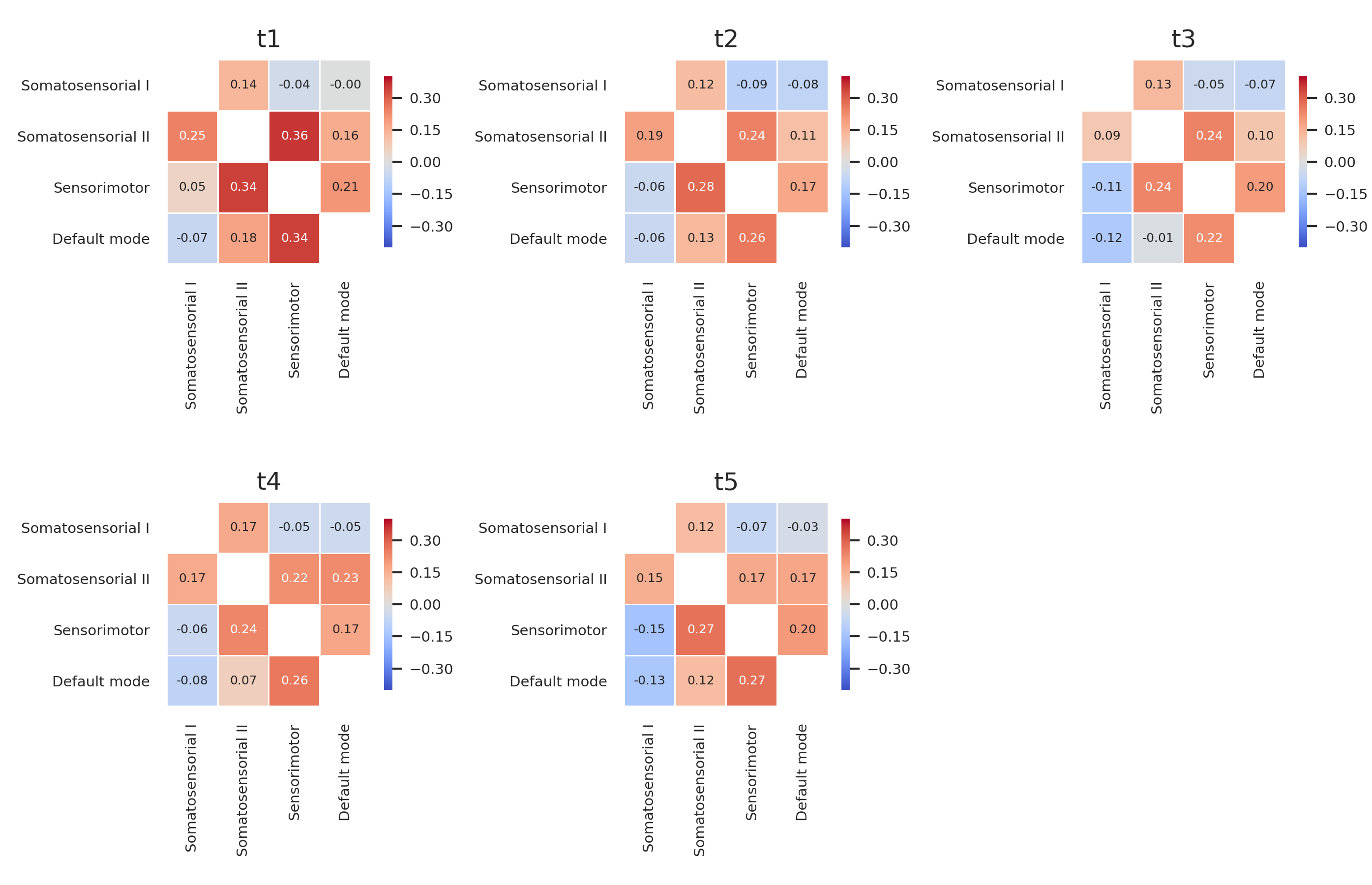

Supplement: FIGURE S2 — Correlation between the time series of each network. Each graph presents the average correlation between the four functional networks for each time point (t1–t5), in the lower triangle the results for the wild type group and in the upper triangle for the TgF344-AD group. [file Image_2.TIF]

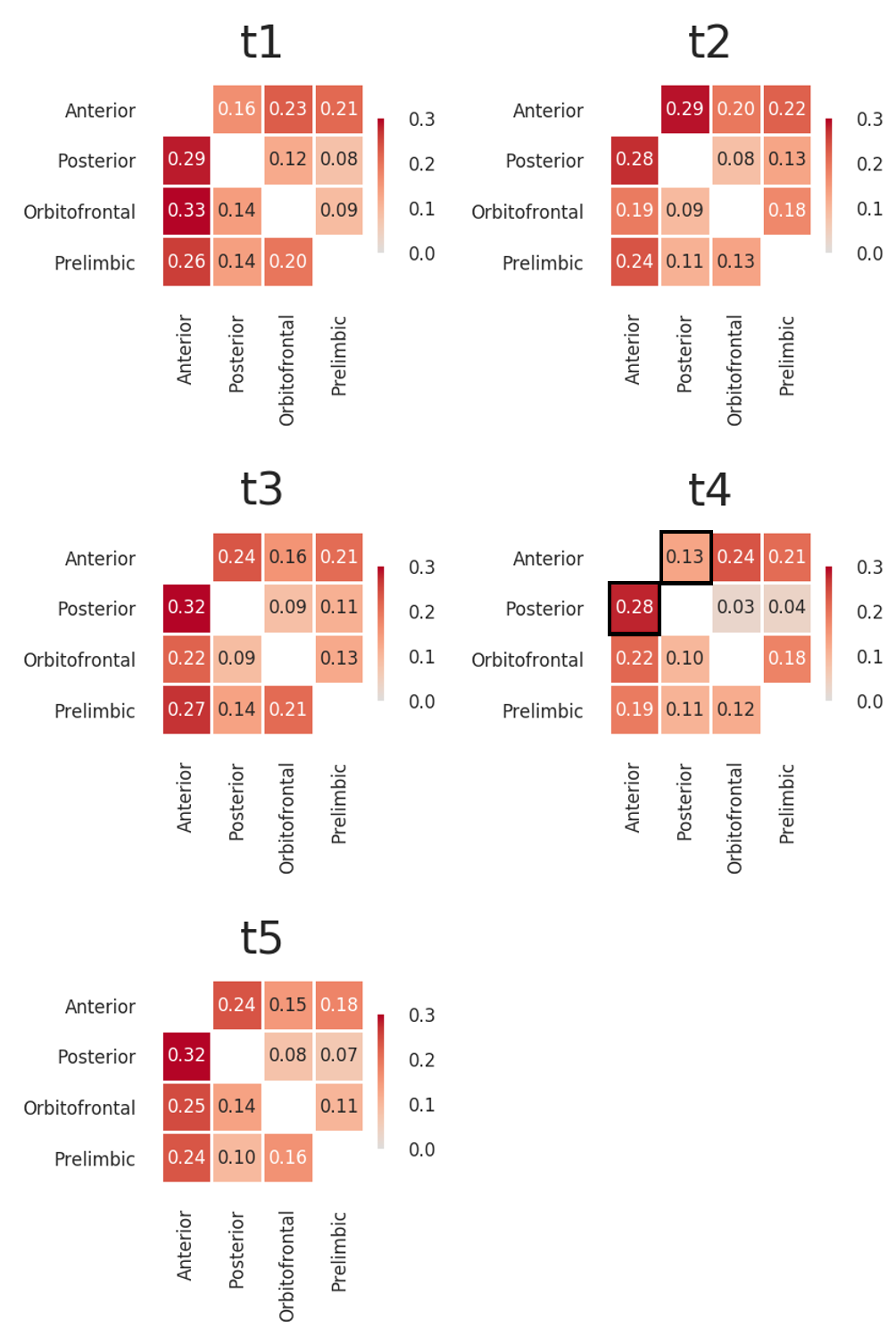

Supplement: FIGURE S3 — Mean connectivity between the four DMN subnetworks (anterior, posterior, orbitofrontal and prelimbic) at each time point (t1–t5). In the lower triangle the results for the wild type group and in the upper triangle for the TgF344-AD group. Black boxes indicate a difference (p < 0.05) between groups. [file Image_3.TIF]

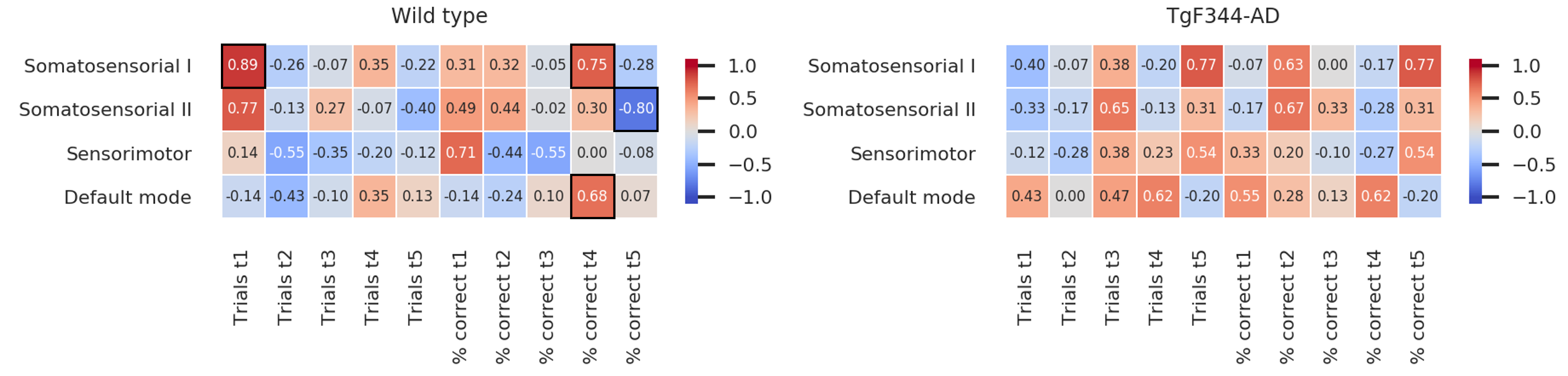

Supplement: FIGURE S4 — Spearman correlation coefficients between each network shape and the number of trials and ratio of correct responses of the DNMS test at the five time-points for the wild type (right column) and the TgF344-AD (left column) groups. Black boxes indicate significant Spearman correlation (p < 0.05). [file Image_4.TIF]

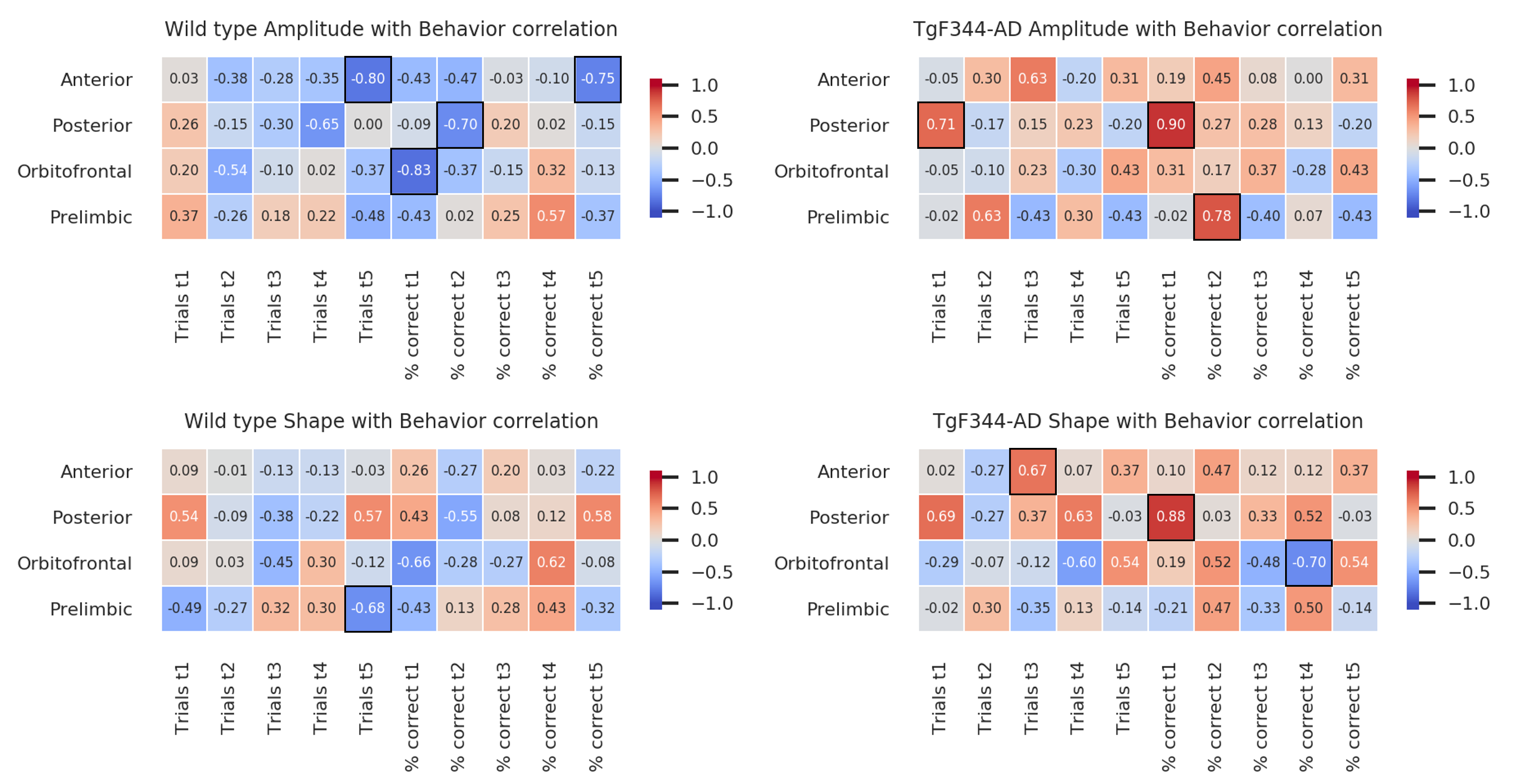

Supplement: FIGURE S5 — Spearman correlation coefficients between DMN subnetworks amplitude (top row) or shape (low row) and the number of trials and ratio of correct responses of the DNMS test at the five time-points for the wild type (right column) and the TgF344-AD (left column) groups. Black boxes indicate significant Spearman correlation (p < 0.05). [file Image_5.TIF]
